# Potential predictive role of gut microbiota to immunotherapy in HCC patients: a brief review

**DOI:** 10.3389/fonc.2023.1247614

**Published:** 2023-08-25

**Authors:** Paola Muscolino, Barbara Granata, Fausto Omero, Claudia De Pasquale, Stefania Campana, Alessia Calabrò, Federica D’Anna, Fabiana Drommi, Gaetana Pezzino, Riccardo Cavaliere, Guido Ferlazzo, Nicola Silvestris, Desirèe Speranza

**Affiliations:** ^1^ Medical Oncology Unit, Department of Human Pathology “G.Barresi”, University of Messina, Messina, Italy; ^2^ Laboratory of Immunology and Biotherapy, Department of Human Pathology “G.Barresi”, University of Messina, Messina, Italy; ^3^ Division of Clinical Pathology, University Hospital Policlinico G.Martino, Messina, Italy; ^4^ Department of Experimental Medicine (DIMES), University of Genoa, Genova, Italy; ^5^ Unit of Experimental Pathology and Immunology, IRCCS Ospedale Policlinico San Martino, Genova, Italy

**Keywords:** hepatocellular carcinoma, advanced, microbiota, immunotherapy, biomarkers

## Abstract

The recent evolution of immunotherapy has revolutionised the treatment of hepatocellular carcinoma (HCC) and has led to new therapeutic standards. The advances in immunotherapy have been accompanied by the recognition of the role of the gut-liver axis in the progression of HCC but also of the clinical relevance of the gut microbiota, which influences host homeostasis but also cancer development and the response to treatment. Dysbiosis, by altering the tumour microenvironment, favours the activation of intracellular signalling pathways and promotes carcinogenesis. The gut microbiota, through their composition and immunomodulatory role, are thus strong predictors of the response to immune checkpoint inhibitor (ICI) treatment as well as an available target to improve ICI efficacy and reduce drug toxicities. In this review we examine the novel role of the gut microbiota as biomarkers in both the diagnosis of HCC and the clinical response to immunotherapy as well as its potential impact on clinical practice in the future.

## 1 Introduction

Hepatocellular carcinoma (HCC) represents 75-85% of all primary liver cancers with a worldwide incidence of 850,000 new cases per year (1). Despite recent progress in the treatment of HCC, 70%-80% of patients are diagnosed with advanced stage disease (2). In the majority of cases, HCC occurs in the context of underlying cirrhosis and chronic inflammation of the liver. The main risk factors for HCC are HBV/HCV infection, heavy alcohol consumption, aflatoxin B1 ingestion, tobacco smoking and non-alcoholic fatty liver disease (NAFLD) caused by obesity and insulin resistance (3).

The gut microbiota comprises roughly a trillion microorganisms, including bacteria, viruses, fungi, and protists, thus constituting a widely diversified micro-ecosystem that is continuously influenced by external factors (1–3). The relationship of the gut microbiota to the immune system and thus to carcinogenesis, including HCC, has been the focus of considerable research attention (4). Some of these studies have postulated an association between HCC and dysregulation of the microbiota, leading to the production of atypical bacterial metabolites, a process known as intestinal dysbiosis (5). The gut-liver axis, through the biliary tract, hepatic portal vein, and biliary secretions, form a pathway by which gut bacteria and their metabolites can translocate to the liver, inducing hepatic inflammation, carcinogenesis, and tumour progression. However, this pathway can also be exploited to enhance the therapeutic response. In fact, the gut microbiome is now seen as playing a key role in the clinical response to ICIs, as assessed by progression-free survival (PFS) and overall survival (OS) (6, 7).

The Barcelona Clinic Liver Cancer (BCLC) staging system classifies HCC according to patient characteristics (performance status, PS) and prognosis (Child-Pugh, alpha-fetoprotein [AFP] level, MELD, ALBI score, tumour size and liver function) (8). Stage 0 (very early stage) to stage B (intermediate stage) HCC is characterised by small tumour size, preserved liver function and PS 0. Treatment strategies are therefore locoregional treatment and include ablation, resection, transplantation and trans-arterial chemoembolisation. However, patients with stage C (advanced stage) HCC, characterised by portal invasion and/or extrahepatic spread, preserved liver function and PS 1–2, will benefit from treatment with immune checkpoint inhibitors (ICIs), discussed in detail below. For patients with stage D (terminal stage) HCC, characterised by any tumour size and end-stage liver function, best supportive care is provided (8).

In this brief review, we focus on the microbiota and its ability to influence the response to ICIs in patients with advanced stage HCC. We examine the differences in the composition of the microbiota between responders and non-responders and how its modulation may be instrumental in obtaining a better therapeutic response.

## 2 ICIs: a new therapeutic strategy in HCC

Although, the molecular alterations in HCC have been extensively studied, the most common mutations are not actionable and only 2.5% of HCC patients have mutations for which target drugs can be used. For the remaining, and indeed the great majority of patients, alternatives are needed (9). In these patients, ICIs, including PD-1 and PD-L1 antibodies, offer an innovative approach as both first-line and second-line therapeutic agents (2).

ICIs have revolutionised cancer therapy and their use has become the standard of care in patients with unresectable or metastatic HCC (10, 11). They include antibodies that target programmed death-1 (PD-1) and programmed death ligand-1 (PD-L1), cytotoxic T lymphocyte antigen-4 (CTLA-4) as well as vascular endothelial growth factor (VEGF). PD-1 is a cell surface protein that is expressed on numerous immune cells and it can be up-regulated after T cell activation; the binding of PD-1 and PD-L1/PD-L2 on target cells suppresses immune cells reactions and causes peripheral tolerance which facilitates tumour growth (12). CTLA-4 is a protein receptor that functions as an immune checkpoint and downregulates immune responses while VEGF is a growth factor that mediates angiogenesis. Additional targets include immunoglobulins, Tim-3 (mucin-domain containing-3), lymphocyte activation gene 3, and transforming growth factor (TGF)-β (13).

In patients with HHC stage C, first-line systemic treatment typically consists of either Atezolizumab (PD-L1 inhibitor) + Bevacizumab (angiogenesis inhibitor, anti-VEGF monoclonal antibody) or Durvalumab (PD-L1 inhibitor) + Tremelimumab (CTLA-4 inhibitor). Patients ineligible for this regimen are treated with Sorafenib (angiogenesis inhibitor) or Lenvatinib (angiogenesis inhibitor) or Durvalumab only. Second-line therapy is based on the previously chosen regimen. For example, Sorafenib may be followed by Regorafenib, an anti-VEFG antibody (if the patient previously tolerated Sorafenib), Cabozantinib (inhibits tyrosine kinases, including VEGF receptors 1, 2, and 3, MET, and AXL), or Ramucirumab (if the AFP level is ≥ 400 ng/mL). Patients initially treated with Atezolizumab + Bevacizumab, or Durvalumab + Tremelimumab, or Lenvatinib or Durvalumab alone should be recruited in clinical trials for further treatment. As third-line treatment, Cabozantinib is the drug of choice but if its use is not possible the patient should be entered into a clinical trial.

The above-mentioned ICIs and others have been or are currently being examined in clinical trials. The anti-PD-L1 antibody Atezolizumab was analysed in IMbrave150, a phase III trial, in combination with the anti-VEGF antibody Bevacizumab vs. Sorafenib in patients with unresectable HCC (1–12). Coprimary endpoints were OS and PFS: OS at 12 months was 67.2% (95% confidence interval [CI] 61.3–73.1) vs 54.6% (95% CI 45.2–64.0) in the Atezolizumab + Bevacizumab and Sorafenib groups respectively. The median PFS was 6.8 months (95% CI 5.7–8.3) and 4.3 months(95% CI 4.0–5.6) respectively. Combination therapy was associated with significantly better OS and PFS than obtained with Sorafenib, resulting in a new standard of care for patients with advanced HCC not amenable to local treatment (14).

The anti-PD-1 antibody Nivolumab was explored in CheckMate 459, an open label phase 3 trial in which Nivolumab (240 mg intravenously every 2 weeks) was compared to Sorafenib (400 mg orally twice daily) as monotherapy in the first-line treatment of patients with advanced HCC. The primary endpoint was OS. Median OS was 16.4 months (95% CI 13.9–18.4) in the experimental arm and 14.7 months in the control arm (95% CI 11.9–17.2). First-line treatment with Nivolumab did not significantly improve OS compared with Sorafenib, but its safety was better (15).

KEYNOTE-224 and KEYNOTE-240 evaluated Pembrolizumab, another anti-PD-1 antibody, in patients who did not tolerate Sorafenib or with disease progression while on the drug. KEYNOTE-240 was a randomised, double-blind, phase III trial assessing Pembrolizumab plus best supportive care (BSC) or placebo plus BSC. The primary endpoints were OS and PFS. At the final analysis, the median OS was 13.9 months (95% CI 11.6–16.0) in the Pembrolizumab group compared with 10.6 months (95% CI 8.3–13.5) in the placebo group. The median PFS was 3.0 months (95% CI 2.8–4.1) and 2.8 months (95% CI 1.6–3.0) respectively. The differences in the OS and PFS between the two groups were not statistically significant (16).

Several other ICIs, including PD-1 (Camrelizumab and Sintilimab), PD-L1 (Durvalumab), CTLA-4 (Tremelimumab) and VEGF (Bevacizumab) antibodies, are being investigated as mono- or combination therapy in ongoing phase III trials in patients with advanced HCC. For example, the ORIENT-32 trial, a randomised, open-label phase 2–3 study, assessed the safety, tolerability and efficacy of Sintilimab (anti PD-1 inhibitor) combined with IBI305 (Bevacizumab biosimilar) vs. Sorafenib as first-line treatment in patients with HBV-associated unresectable HCC. The primary endpoints were OS, PFS, safety and tolerability. OS was higher in the experimental arm than in the control arm (not reached vs. 10.4 months). In the Sintilimab + IBI305 arm, PFS was higher than in the control arm (4.6 months [95% CI 4.1–5.7] vs. 2.8 months [95% CI 2.7–3.2]). The acceptable safety and tolerability of this ICI regimen recommend it as a new and promising therapeutic strategy for patients with advanced HCC (17).

A focus of the Gastrointestinal Cancers Symposium of the American Society of Clinical Oncology (ASCO GI, 2023) was the treatment of HCC, especially with immunotherapy agents. Among the reported results were those of the HIMALAYA trial, a phase III, global, open-label study in which patients were randomised into three arms: (i) a combination arm (STRIDE: single Tremelimumab regular interval Durvalumab), consisting of Tremelimumab (300 mg one dose) + Durvalumab (1500 mg every 4 weeks), (ii) Durvalumab (1500 mg every 4 weeks), or (iii) Sorafenib (400 mg twice daily). The study demonstrated the superiority of the STRIDE regimen based on a median OS of 16.4 months (95% CI 14.16–19.58) vs. 13.7 months (95% CI 12.25–16.13) in patients treated with Sorafenib alone. In the STRIDE arm, the survival rate at 36 months was 30.7% (95% CI 25.8–35.7) compared to 20.2% (95% CI 15.8–25.1) in the Sorafenib group. The OS of patients who received Durvalumab as monotherapy was non-inferior to that of patients treated with Sorafenib alone. Reported adverse events, which were of all grades, were minor in the STRIDE-treated arm compared to the Sorafenib-treated arm. The demonstrated efficacy of the STRIDE regimen vs. Sorafenib will lead to a new first-line treatment option in patients with unresectable HCC (18).

Clinical trials concerning the use of ICIs in patients with HCC are summarised in **Table 1**.

**Table 1:** Immune checkpoint inhibitors (ICIs) and hepatocellular carcinoma (HCC), main clinical trials.

CLINICAL TRIAL	TREATMENT	PRIMARY ENDPOINT	RESULTS	SECONDARY ENDPOINT	RESULTS
**CHECKMATE 459**	Nivolumab (371)vs Sorafenib (372)	**OS**	**mOS** (months) 16.4 vs 14.7	**ORR, mPFS**	**ORR** 15% *vs 7%* **mPFS** (months) 3.7 vs 3.8
**KEYNOTE 24**0	Pembrolizumab + BSC (278) vs Placebo + BSC (135)	**OS, PFS**	**mOS** (months) 13.9 vs 10.6 **mPFS** (months) 3 vs 2.8	**ORR, DOR, QoL**	**ORR** 27,3% vs 11,9% **DOR** 87,6% vs 59,1% **QoL**1 1.2 vs 3.6
**HIMALAYA**	STRIDE regimen ( Single Tremelimumab Regular Interval Durvalumab) (389) vs Durvalumab alone (389) vs Sorafenib alone (389)	**OS**	**mOS** (months) STRIDE regime 16.43 vs Sorafenib 13.77	**mOS, mPFS, ORR, DCR, DOR**	**mOS** (at 36 months) 16.56 vs 13.77 **mPFS** 3.78 vs 4.07 (STRIDE vs Sorafenib) 3.65 vs 4.07 (Durvalumab vs Sorafenib) **TTP (**months): 5.4 vs 3.8 vs 5.6 **ORR** 20.1% vs 17,0% vs 5,1% **DCR** 3.1% vs 1.5% vs 0% **mDOR** (months) 22.3 vs 16.8 vs 18.4
**ORIENT-32**	Sintilimab + IBI305 (380) vs Sorafenib (191)	**Safety and tolerability, mOS, mPFS**	Acceptable safety profile **mOS (**months): NR vs 10.4 **mPFS** (months): 4.6 vs 2.8		
**IMBRAVE 150**	Atezolizumab + Bevacizumab (336) vs Sorafenib(165)	**OS, PFS**	**OS** 67% vs 54.6% **mPFS(**months) 6.8 vs 4.3	**ORR, DOR, QoL**	**ORR**: 27.3% vs 11.9% **DOR:** 87.6% vs 59.1% **QoL:** 11.2 vs 3.6

OS, overall survival; PFS, progression-free survival; ORR, objective response rate; DCR, disease control rate; DOR, duration of response; TTP, time to progression; TKI, tyrosine-kinase inhibitors; BSC, best supportive care; NR, not reached; QoL, quality of life-median time to deterioration.

Despite the therapeutic gains achieved with ICIs, they are a non-curative treatment, unlike surgical approaches or transplantation in the small proportion of patients with non-advanced disease (12).

## 3 The influence of the microbiota on the tumour response to ICIs

Recent clinical trials reported that ICI-responder patients were distinguished from non-responders by a microbiota of favourable composition, i.e., capable of inducing antitumour immune responses (19–21). For example*, Akkermansia muciniphila*, abundant in responder patients, induced a type-1 antitumour response that included the stimulation of interleukin (IL)-12 secretion by dendritic cells (DCs), which in turn increased the recruitment of memory CD4+ T cells. These patients had a good clinical outcome and a longer PFS (22).

A positive influence on ICI treatment was also determined for *Bacteroides fragilis*, through its activation of interferon (IFN)-γ- producing CD4+T cells and DCs (23, 24). Both single species, such as *Bifidobacterium *(25), and a mixture of species, including those of the genera *Bacteroides, Ruminococcaceae, Eubacterium* and *Fusobacterium*, were shown to enhance the efficacy of anti-PD-L1 and anti-PD-1, by eliciting strong IFNγ+ CD8+ T cell responses (19–26). The enrichment of these species in ICI responders with liver cancer (27, 28) suggests their ability to promote an antitumour response via the activation of CD8+ T cells, which are pivotal in controlling HCC outgrowth (29).

Recent studies indicated that not only commensal bacteria but also their metabolites can improve the efficacy of cancer therapy, by inducing antitumour immune responses. Metabolomic approach investigated the set of metabolites expressed by a particular biological system. Analyses of the metabolome derived from serum or the faecal microbiome may lead to the identification of predictive biomarkers that can be used to predict a long-term response to immunotherapy. For example, microbiome-derived short-chain fatty acids (SCFA) modulate CD8+ T cell responses in anti-PD-1 responder patients and their presence is associated with a longer PFS (30, 31). In a model of pancreatic cancer, SCFA strongly activated the effector functions of CD8+ T cells in adoptive cell therapy, thus increasing anti-tumour reactivity, which improved the therapeutic outcome (31). This finding suggests that the administration of SCFA or bacteria producing SCFAs can improve the effectiveness of the adoptive CD8+T cell therapy currently used in HCC patients. In a mouse model of HCC, the administration of SCFA in combination with anti-PD-1 improved the therapeutic efficacy of the latter, by reducing pro-tumourigenic IL-17 release and in turn suppressing tumour growth (32). The bacterial metabolite,inosine, mainly derived from *Bifidobacterium species*, also positively impacted ICI efficacy, by inducing both the activation of CD4+ T and CD8+T cells and robust IFN-γ release (33).

Conversely, non-responder patients are affected by the profound dysbiosis that prevent the activation of type 1 immune response and promote, instead, immunosuppressive response. In non-responder HCC patients, IL-10 production by DCs lead to the expansion of Tregulatory cells that suppress anti-tumor immune response (34–36).

**Figure 1:**
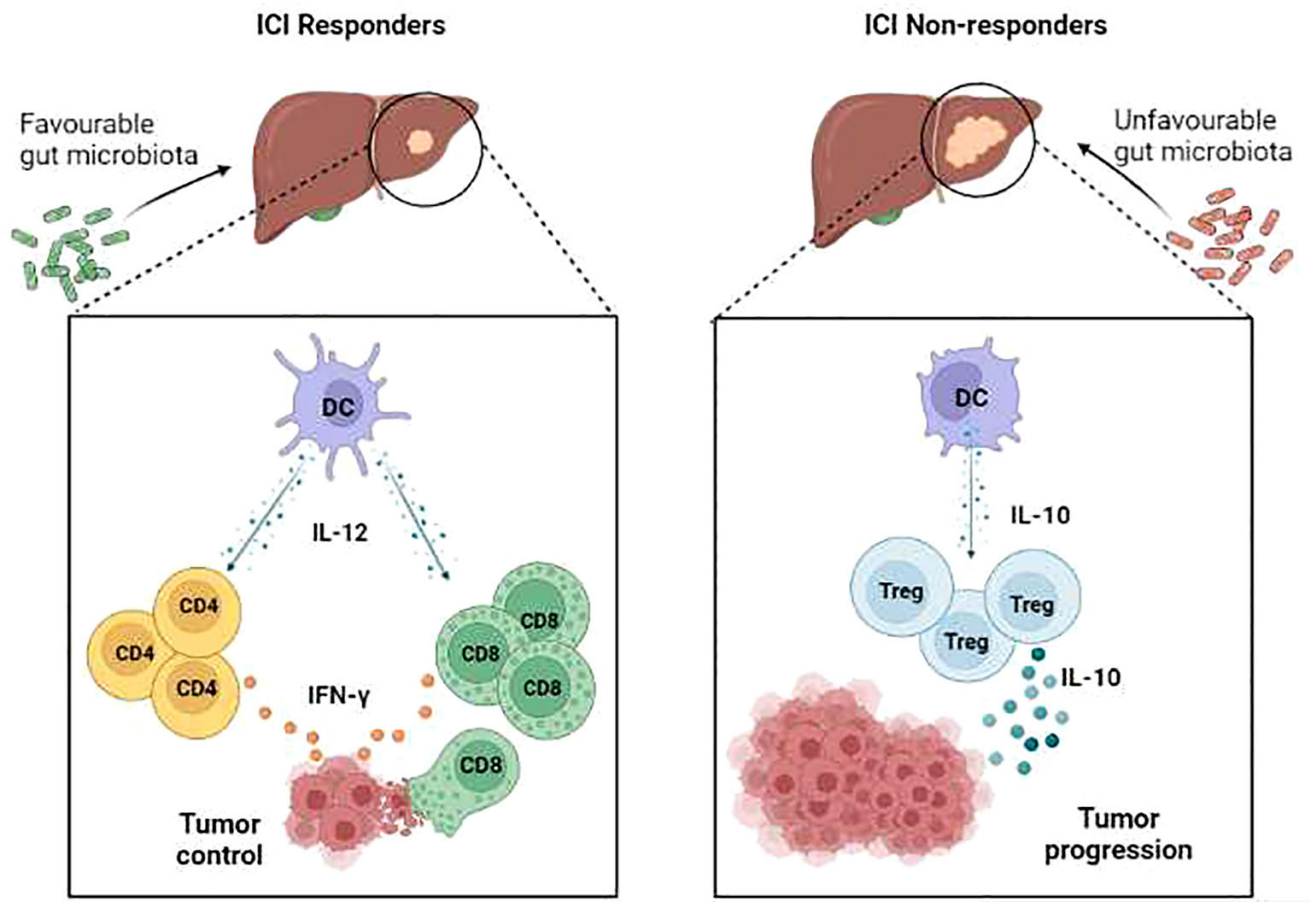
Immune cell responses in ICI responder vs ICI non-responder patients with HCC. In patients who are ICI responders, a favourable microbiota is associated with interleukin (IL)-12-producing dendritic cells (DC) that support activation of both Th1 immune responses and cytotoxic T lymphocyte (CTL)-mediated cytotoxicity, resulting in the effective control of tumour growth. Conversely, in patients who are ICI non-responders, a dysbiotic microbiota is associated with tolerogenic DC that drive the expansion of immunosuppressive IL-10-producing T regulatory cells, thus supporting tumour progression.

These observations highlight the need for interventional strategies aimed at supporting a microbiota able to promote an antitumour response and prevent immune resistance (**Figure 1**).

## 4 Differences between the microbiome in responders and non-responders

Among patients with advanced HCC, the differences between responders and non-responders to ICIs, specifically ICIs targeting anti-PD1 and anti-PD-L1, are considerable. The contribution of the microbiome to this difference is supported by several studies in which the microbiome of faecal samples taken at baseline (T0) and during treatment, usually 2 months after the start of therapy, was sequenced. In those studies, the sequencing targets were the V3 and V4 regions of 16s RNA and the whole genome, determined from stool samples. In some of those studies, the patient population comprised patients with advanced HCC who had already undergone chemotherapy (e.g. with Sorafenib) (9–37).

The results were fairly unanimous. In the study of Min-Woo Chung et al., patients were divided into responders and non-responders according to the RECIST 1.1 criteria. Patients in the responders group had higher relative abundances of *Citrobacter freundii, Azospirillum*sp. and *Enterococcus durans*, while among non-responders the prevailing species were *Dialisterpneumosintes, Escherichia coli, Lactobacillus reteri, Streptococcus mutans, Enterococcus faecium, Streptococcus gordonii, Veillonellaatypica, Granulicatella*sp. and *Trichuristrichiura.* Furthermore, an unbalanced ratio of *Firmicutes* to *Bacteroidetes* was shown to be associated with a reduced response to treatment, and a high ratio of *Prevotella to Bacteroides* with a good response to Nivolumab (37).

Mao Jinzhu et al. also reported different clinical responses in patients with advanced hepato-biliary tumours treated with anti-PD-1. Of the 65 patients included in the study, 30 had HCC and 35 had biliary tract cancer. The treatment outcomes were classified according to the RECIST 1.1. criteria: 20 patients achieved a partial response, 28 had stable disease and 17 had disease progression. Stool samples were collected at baseline from all patients as well as from eight patients during treatment (four with and four without clinical benefit). Patients with a clinical benefit from treatment had higher levels of *Bacterioides and Firmicutes* while in those with NCB higher levels of *Proteobacteria* and of bacteria of the order *Veillonellales* were determined. The statistical analysis showed a reduction in PFS and OS in the group with a greater relative abundance of *Veillonellaceae *but a higher PFS and a higher OS in the group with a greater relative abundance of *Lachnospiraceae*, *Erysipelotrichaceae bacterium-GAM147, Ruminococcuscallidus*, *Alistipes*sp. *Marseille-P5997* and *Bacteroides zoogleoformans.* The commensal species *Lachnospiraceae-GAM79 and Erysipelotrichaceae-GAM147* are butyrate-producing, protective strains that, through the production of TGF-β and IL-10, improve epithelial barrier function; they are also involved in bile acids metabolism. In faecal samples collected during treatment, the metagenomic analysis showed that, in patients with a good response to treatment, the microbiome population remained stable throughout treatment, while in patients with a poor response microbial diversity declined (27).

Zheng et al. examined the microbiome in patients with HCC progression after Sorafenib therapy. Those patients, with HCC stage C, were treated with the anti-PD-1 agent Camrelizumab, with faecal samples taken at baseline (day 0), after 1 week and every 3 weeks during therapy until disease progression. Genomic analysis showed that responders had a higher microbiome population richness than non-responders. The difference between the bacterial population at the start of treatment and during treatment was investigated as well and showed high relative abundances of *Firmicutes, Bacteroides* and *Proteobacteria* in both responders and non-responders but a higher concentration of *Akkermansia muciniphila* and *Ruminococcaceae* in responders. In non-responders, there was an evident change in the bacterial population during treatment, including an increase in *Proteobacteria*,mainly *E. coli*, from week 3 until week 12, at which point the first species became predominant. By contrast, in responders *Proteobacteria* were mainly represented by *Klebsiella pneumoniae*. This study showed that the microbiome changes in relation to treatment and the potential utility of microbiome analysis in predicting the treatment response (28).

Li et al. demonstrated a correlation between the oral and gut microbiome and PFS. Patients with a higher level of *Faecalibacterium* had a longer PFS than those with a higher level of *Bacteroidales*. Thus, a higher relative abundance of *Faecalibacterium *may be a feature of responders and a higher relative abundance of *Bacteroidales*a feature of non-responders (2). By contrast, the study of Shen et al., which enrolled 36 patients with advanced HCC, did not reveal any difference between responders and non-responders either at baseline or in relation to the administered ICI type. However, the microbiota is strongly affected by external factors (environmental, dietary, sex, medication), such that a large study population together with different methods of sample storage and analysis can compromise the study results.

**Figure 2:**
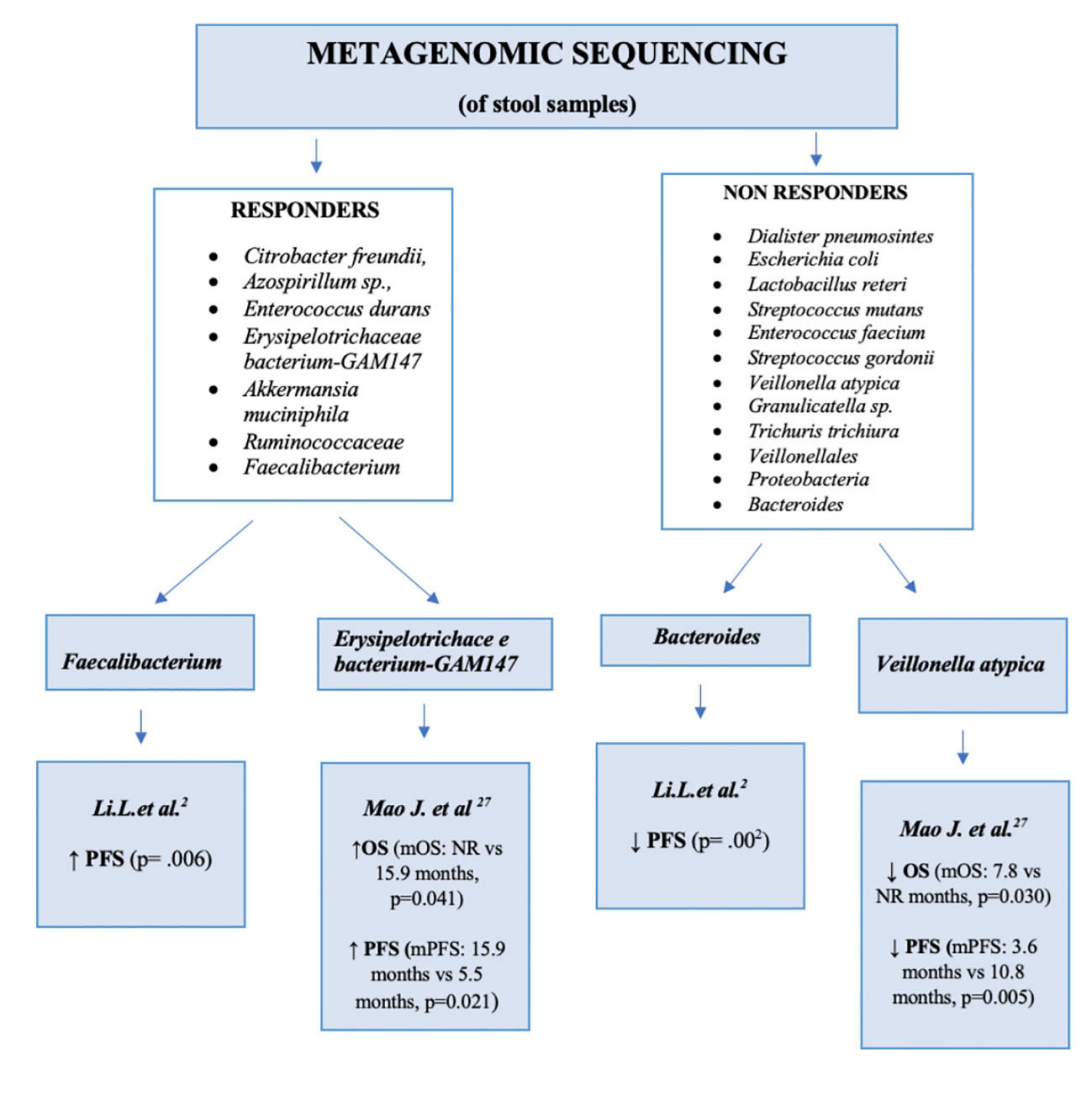
Differences in the microbiota: between responders and non-responders. PFS, progression-free survival; OS, overall survival. mPFS, median progression-free survival; mOS, median overall survival; NR, not reached.

**Figure 2** compares the main bacterial species found in the microbiota of responders and non-responders.

## 5 Future perspectives

**Figure 3:**
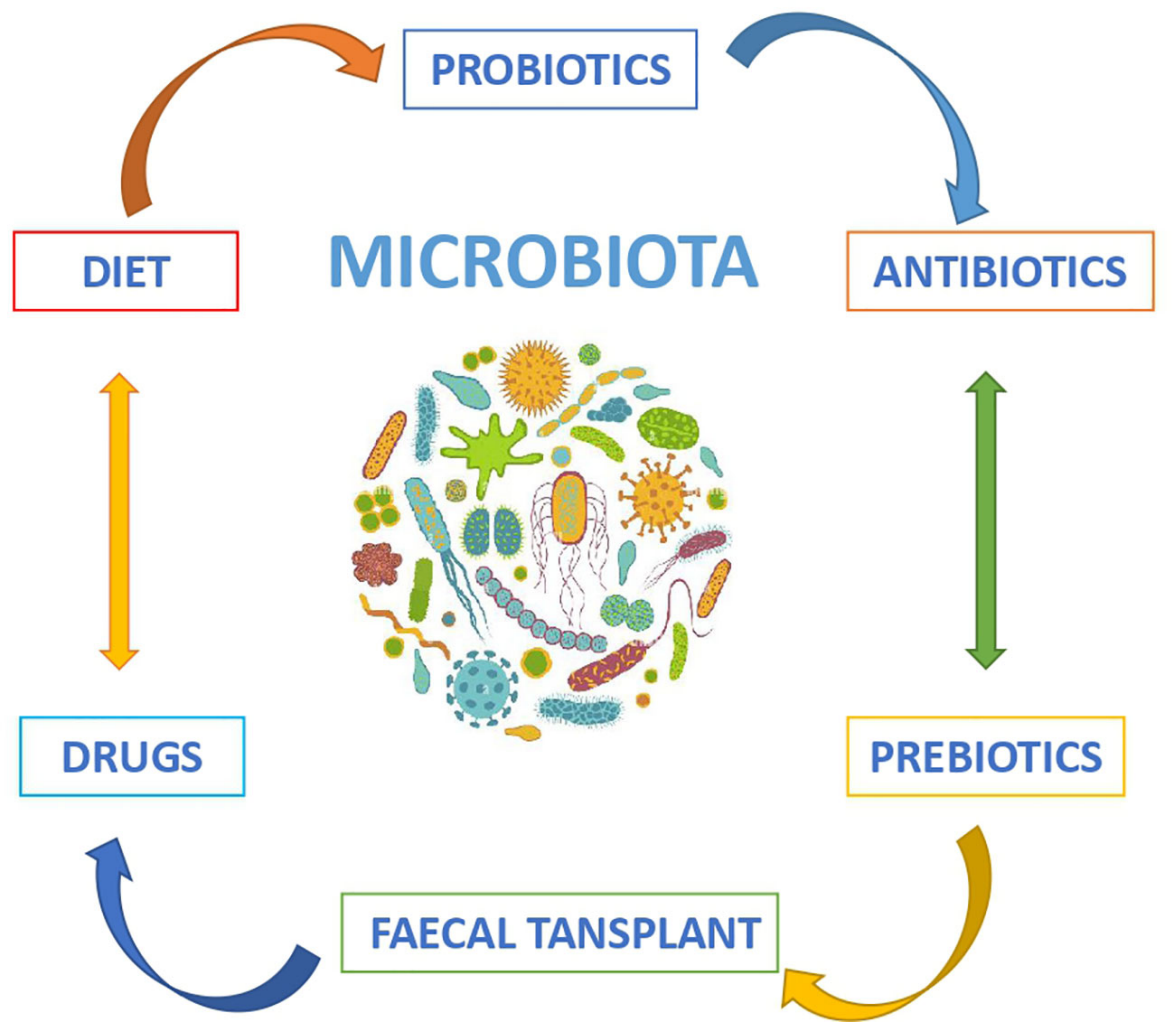
Modulators of the intestinal microbiota.

Recent studies on the role of the microbiome in HCC treated with ICIs suggest microbiome modulation as a novel therapeutic approach to indirectly modify the clinical response to current treatment. The options for microbiome modulation include diet, probiotics, prebiotics, antibiotics and faecal transplant (**Figure 3**) (4).

Probiotics are living microorganisms that, following their ingestion, colonise the intestine and provide healthy benefits, generally by improving or restoring the gut microbiota. Traditional probiotics include *Bifidobacterium* and *Lactobacillus* whereas more recent next generation probiotics contain butyrate-producing members of *Clostridium* and *Akkermansia muciniphila*, a mucin degrader. Oral introduction of these bacterial species may confer anti-inflammatory and anti-cancer effects through the production of metabolites and the modulation of immune cells. Probiotics may thus intervene in the pathogenesis of HCC, by acting on gene expression and by counteracting the cancer-promoting effects of viral infections as well as the lipotoxicity of NAFLD. A similar mechanism of action and similar results derive from the use of prebiotics, which are non-digestible food ingredients that stimulate the growth and/or activity of bacteria in the colon to benefit the host (1). The microbiome can also be modulated by transplanting bacteria from a donor to a host. In faecal microbial transplantation (FMT) a ‘physiological’ microbiome from carefully selected, healthy individuals is transferred (4). A FMT variant is microbial ecosystem therapeutics (MET), in which the transplanted material is a defined mixture of pure living cultures of intestinal bacteria isolated from a stool sample of a healthy donor (38). The microbiota can be transferred via endoscopy, enema or oral capsules, but so far there is no agreement about the optimal frequency, dose, and duration of FMT. Thus far, FMT has been used to eradicate *Clostridium difficile* infection whereas in oncology the focus has mainly been on the anti-inflammatory effects of the transplant in order to prevent chronic liver disease and its progression to HCC.

The alterations in the tumour microenvironment in response to changes in the microbiome, and particularly with respect to immune cell infiltration and gene expression, have been examined as well (4). For example, in mouse models and in patients receiving immunotherapy, *Ruminococcaceae* family transplantation was shown to enhance antitumour immunity, by increasing tumour infiltration by IFN-γ+CD8+ T cells (19–27). These early results provide the basis for in depth studies of the role of FMT in immune checkpoint modulation. Although the focus of FMT studies has been on tumour types other than HCC, their results may nonetheless be relevant to HCC, as both animal and human studies have shown that FMT in patients responding to immunotherapy improves the host response to the immunotherapy itself (5). Studies in patients with other tumour types have also shown that the clinical response may be influenced by antibiotics/proton pump inhibitors as modulators of the microbiome (38).

While the chronic use of antibiotics is known to lead to gut dysbiosis and may disrupt the potential benefit of ICIs, rifaximin, a non-systemic antibiotic with low gastrointestinal absorption used to prevent hepatic encephalopathy, was shown to induce the growth of beneficial bacteria such as *Bifidobacterium*, *Faecalibacterium* and *Lactobacillus*, and to exert an anti-inflammatory effect. However, while this use of rifaximin may be promising, it has yet to be tested in preclinical phase models of HCC. *David J. Pinato et al.* analyzed in a retrospective study through an FDA database across nine multicenter studies, the association between antibiotics and adverse cancer outcomes in patients treated with immunotherapy or targeted therapy for hepatocellular carcinoma. Antibiotic therapy use within 30 days before or after treatment initiation was related to overall survival (OS) and progression-free survival (PFS) by therapeutic regimens before and after inverse probability of treatment weighting (IPTW). Of 4,098 patients with unresectable/metastatic HCC, those receiving antibiotic therapy (n = 620, 15%) was associated with shorter median PFS (3.6 months in ATB-exposed versus 4.2 months; hazard ratio [HR] 1.29; 95% CI 1.22, 1.36) and OS (8.7 months in ATB-exposed versus 10.6 months; HR 1.36; 95% CI 1.29, 1.43). In IPTW analyses, antibiotic (ATB) exposure was associated with shorter PFS in patients treated with ICI (HR 1.52; 95% CI 1.34, 1.73), TKI (HR 1.29; 95% CI 1.19, 1.39), and placebo (HR 1.23; 95% CI 1.11, 1.37). The clinical study showed that early antibiotic treatment was associated with worse outcomes not only in patients treated with immune checkpoint inhibitors, but also in those treated with tyrosine kinase inhibitors and placebo (39). Among the drugs that influence the intestinal microbiota, metformin should also be mentioned, as it is associated with a reduction in the incidence of HCC, through an anti-inflammatory action possibly mediated by an increase in the abundances of *Bifidobacterium* and *Akkermansia*. Similar results have been reported with aspirin and statins (40).

## 6 Conclusions

Further advances in ICIs and other forms of immunotherapy with high levels of efficacy and safety in patients with advanced HCC can be expected. The microbiota has an important influence on the response to treatment with ICIs and may therefore serve as a predictor of responder and non-responder patients.

## Author contributions

PM and BG initiated the study, wrote the manuscript and constructed the figures; FO, DS, AC and GP wrote the manuscript and constructed the figures; VR, RC, SC, FD and FD’A wrote the manuscript; GF and CP wrote and revised the manuscript. NS and DS ideated the study, supervised all aspects and wrote and revised the manuscript. All authors have read and agreed to the published version of the manuscript.
